# Spatiotemporal change analysis of long time series inland water in Sri Lanka based on remote sensing cloud computing

**DOI:** 10.1038/s41598-021-04754-y

**Published:** 2022-01-14

**Authors:** Jianfeng Li, Jiawei Wang, Liangyan Yang, Huping Ye

**Affiliations:** 1 Institute of Land Engineering and Technology, Shaanxi Provincial Land Engineering Construction Group Co., Ltd., Xi’an, 710021 China; 2 State Key Laboratory of Resources and Environmental Information System, Institute of Geographic Sciences and Natural Resources Research, Chinese Academy of Sciences, Beijing, 100101 China; 3 Shaanxi Provincial Land Engineering Construction Group Co., Ltd., Xi’an, 710075 China; 4 Key Laboratory of Degraded and Unused Land Consolidation Engineering, The Ministry of Natural Resources, Ltd., Xi’an, 710021 China; 5 Shaanxi Provincial Land Consolidation Engineering Technology Research Center, Ltd., Xi’an, 710021 China

**Keywords:** Ecology, Hydrology, Limnology

## Abstract

Sri Lanka is an important hub connecting Asia-Africa-Europe maritime routes. It receives abundant but uneven spatiotemporal distribution of rainfall and has evident seasonal water shortages. Monitoring water area changes in inland lakes and reservoirs plays an important role in guiding the development and utilisation of water resources. In this study, a rapid surface water extraction model based on the Google Earth Engine remote sensing cloud computing platform was constructed. By evaluating the optimal spectral water index method, the spatiotemporal variations of reservoirs and inland lakes in Sri Lanka were analysed. The results showed that Automated Water Extraction Index (AWEI_sh_) could accurately identify the water boundary with an overall accuracy of 99.14%, which was suitable for surface water extraction in Sri Lanka. The area of the Maduru Oya Reservoir showed an overall increasing trend based on small fluctuations from 1988 to 2018, and the monthly area of the reservoir fluctuated significantly in 2017. Thus, water resource management in the dry zone should focus more on seasonal regulation and control. From 1995 to 2015, the number and area of lakes and reservoirs in Sri Lanka increased to different degrees, mainly concentrated in arid provinces including Northern, North Central, and Western Provinces. Overall, the amount of surface water resources have increased.

## Introduction

**Figure 1:**
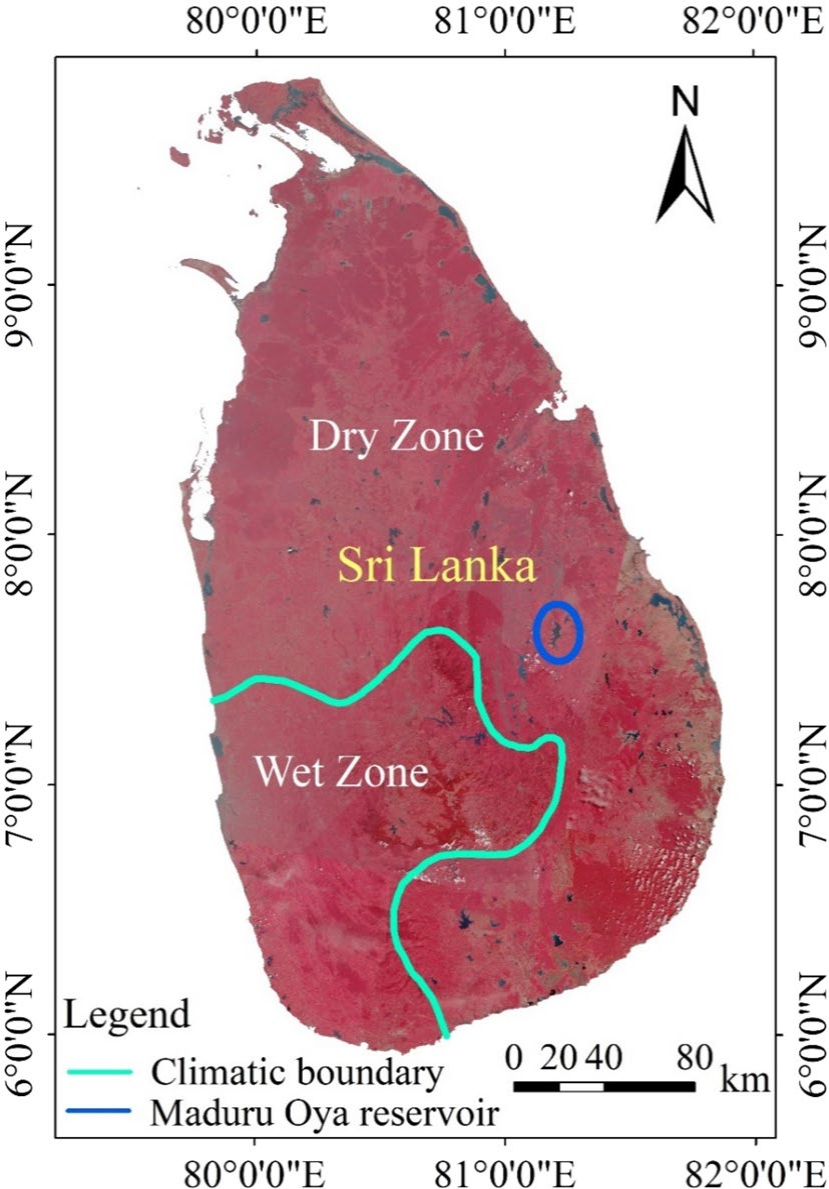
Location of the study area. The map was generated by geospatial analysis of ArcGIS software (version ArcGIS 10.3; http://www.esri.com/software/arcgis/arcgis-for-desktop).

As a hub connecting the Asia-Africa-Europe sea route, Sri Lanka has important economic and geographical significance. Sri Lanka has a tropical monsoon climate. Due to the high mountains in the central part of the country blocking the warm and humid southwest monsoon, only part of the southwestern region of the country experiences humid climate due to frontal rain (Wet Zone, Fig. 1), while most of the other areas are arid (Dry Zone, Fig. 1)^1^. There are evident dry and rainy seasons in the arid areas. Additionally, the temporal and spatial distribution of water resources in the island is highly uneven, which results in a serious seasonal water shortage and a significant fluctuation in the surface water area^2–5^. Because of the unreasonable use of water resources, most of the limited water resources are polluted, which causes serious waterborne diseases in Sri Lanka. Therefore, it is of great significance to study the spatiotemporal variation of inland lakes and reservoirs in Sri Lanka, which can provide a scientific basis for the protection, management, and planning of water resources.

With the rapid development of aerospace technology in recent years, remote sensing technology provides advanced means for many fields such as resource investigation, climate monitoring and global change research^6^. Since the seventies and eighties of the twentieth century, remote sensing technology has been applied for the dynamic monitoring of surface water^4, 7, 8^. Compared with traditional measurement methods, remote sensing can provide real-time, dynamic, and low-cost water image information^9^. There are usually two methods used for extracting the water boundaries: (1) the edge detection method, which is used to extract the surface water boundary of the image directly and (2) the second method, in which water area is extracted first, and then a certain algorithm is used to transform the boundary of the extracted water area^10^. The latter method is most commonly used in studies, among which the spectral water index is the most widely used method for water extraction^10^. At present, the most common spectral water index methods are Normalized Difference Water Index (NDWI)^11^, Modified Normalized Difference Water Index (MNDWI)^12^, Enhanced Water Index (EWI)^13^, Water Index (WI)^14^, and Automated Water Extraction Index (AWEI_sh_)^15^. In general, water bodies are extracted from spectral water index images by setting the threshold value as zero^11, 12^. However, in practical applications, the multispectral images of sensors have various characteristics in different regions and times, which influences the extraction results^16^. Moreover, in Sri Lanka, the water environment is complex; hence, the determination of the threshold has a vital impact on the extraction results. This study attempted to use the OTSU^17^ algorithm to determine the threshold for water extraction. The OTSU^17^ algorithm is an extensively used dynamic threshold determination method that aims to maximise class variance and dynamically determines the threshold for each scene, without having an effect on the image brightness and contrast^18^. With the development of cloud computing and big data technology in recent years, remote sensing cloud platforms such as the Google Earth Engine^19^ and Pixel Information Expert Engine (PIE-Engine)^20^ are now available. These changes have broken the original norm of "image download-pre-processing-model building-result analysis" in remote sensing application research. With the strong computation and storage capacity of the cloud platform, batch analysis and image calculation are performed by calling the Application Programming Interface (API) online, which greatly improves the speed and efficiency of the achievement transformation^21–23^.

At present, there are relatively few studies that have performed the complete process of water extraction utilizing remote sensing cloud computing, and the research on the spatiotemporal variation of inland water in Sri Lanka is still in the blank. In this study, we analysed the spatiotemporal variation of inland water in Sri Lanka, which has not been studied extensively based on a remote sensing cloud computing platform and Landsat-5/8 images; a rapid extraction model of surface water was constructed, and the optimal spectral water index method was determined by a water extraction accuracy test, for obtaining the spatiotemporal variation analysis of typical reservoirs and inland lakes and reservoirs in Sri Lanka.

## Materials and methods

### Study area

Sri Lanka, an island in the Indian Ocean, is located to the south of the Indian sub-continent. It is 432 km long from north to south and 224 km wide from east to west, and covers an area of 65,610 km^2^ ^24^. The average annual rainfall ranges from less than 1000 mm on the southeast coast to more than 4500 mm on the western slope of the plateau^25^. During the monsoon season, there is a short dry season in January and February in the wet zone, with plenty of rain in the remaining months. In the dry zone, there are evident wet (October to February) and dry seasons. Figure 1 shows the location of the study area.

### Data

The Landsat satellite series were released by the United States Geological Survey (USGS) in 1972 and have been in operation for more than 40 years^26^. As a result, it has accumulated rich remote sensing archived data; additionally, it offers the ability to cover the same area repeatedly for a short period of time^27^, and therefore, it can be applied to the analysis of temporal and spatial variation of surface water^28^. The Landsat-5 and Landsat-8 images in this study were collected from the "LANDSAT/LT05/C01/T1_SR" and "LANDSAT/LC08/ C01/T1_SR" datasets provided by the Google Earth Engine (https://earthengine.google.com), amounting to a total of 47 scenes. These two datasets were atmospherically corrected, and the resolution of the multispectral band was 30 m. The Landsat-8 image covering the east-central part of Sri Lanka in June 2014 (Path/Row: 140/055) was selected for comparing the results from water extraction algorithms. The five Landsat-5 images and two Landsat-8 images from 1988 to 2018 (Path/Row: 141/055) were used to analyse the inter-annual variation of typical reservoir; most of the images were concentrated in August with an interval of 4–6 years. The twelve Landsat-8 images in 2017 were used to analyse the intra-annual variation of a typical reservoir. The spatiotemporal variation analysis of inland lakes and reservoirs, including nine Landsat-5 images in 1995, nine Landsat-5 images in 2005, and nine Landsat-8 images in 2015, all of which were concentrated in February.

### Spectral water index method

The spectral water index method primarily utilizes the differences in the spectral characteristics of water bodies in different bands, which is constructed by calculating the ratio of the high reflectivity band to the high absorptivity band, and then the water information is extracted by threshold segmentation^29^. Using the spectral water index method enables the enhancement of image features, reduces the influence of the environmental conditions around the water, and increases the difference between water and other features. The calculation formulas of NDWI^11^, MNDWI^12^, EWI^13^, WI^14^ and AWEI_sh_^15^ are as follows:

1 $${\text{NDWI}} = \frac{{\rho_{Green} - \rho_{NIR} }}{{\rho_{Green} + \rho_{NIR} }}$$

2 $${\text{MNDWI}} = \frac{{\rho_{Green} - \rho_{SWIR} }}{{\rho_{Green} + \rho_{SWIR} }}$$

3 $${\text{EWI}} = \frac{{\rho_{Green} - \rho_{NIR} - \rho_{SWIR} }}{{\rho_{Green} + \rho_{NIR} + \rho_{SWIR} }},$$

4 $${\text{WI}} = \left\{ {\begin{array}{*{20}c} {0,} & {if\;\max \rho_{VIS} \le {\text{max}}\rho_{SWIR} } \\ {1,} & {if\;\max \rho_{VIS} > {\text{max}}\rho_{SWIR} } \\ \end{array} } \right.$$

5 $${\text{AWEI}}_{sh} = \rho_{Blue} + 2.5 \times \rho_{Green} - 1.5 \times \left( {\rho_{NIR} + \rho_{SWIR1} } \right) - 0.25 \times \rho_{SWIR2}$$

In the formula, $\rho_{Blue}$, $\rho_{Green}$, $\rho_{NIR}$, $\rho_{SWIR}$, $\rho_{VIS}$ are the reflectivity of remote sensing images in blue, green, near-infrared, shortwave-infrared and visible light bands, respectively.

### OTSU algorithm

OTSU^17^ mainly divides the grey value of the image into two parts based on clustering, so that each part has a minimum grey difference, and the difference between the two parts is the largest. By calculating the variance, we can find a more appropriate grey level for division. The process of calculating the optimal threshold t of the OTSU algorithm is as follows:

6 $$\left\{ {\begin{array}{*{20}l} {\delta^{2} = P_{nw} \cdot \left( {M_{nw} - M} \right)^{2} + P_{w} \left( {M_{w} - M} \right)^{2} } \hfill \\ {M = P_{nw} \cdot M_{nw} + P_{w} \cdot M_{w} } \hfill \\ {P_{nw} + P_{w} = 1} \hfill \\ {t = Arg \mathop {\max }\limits_{x \le t \le y} \left\{ {P_{nw} \cdot \left( {M_{nw} - M} \right)^{2} + P_{w} \left( {M_{w} - M} \right)^{2} } \right\}} \hfill \\ \end{array} } \right.$$

$\delta$ is the inter-class variance of non-water and water; $P_{nw}$ and $P_{w}$ are the possibility that a single pixel belongs to non-water and water; $M_{nw}$ and $M_{w}$ are the average grey levels of non-water and water pixels, respectively; and $M$ is the average grey level of the image pixel. The OTSU algorithm adaptively determined the threshold values for all the spectral water indices of NDWI, MNDWI, EWI, and AWEI_sh_.

### Rapid extraction model of surface water based on google earth engine

Google Earth Engine is a cloud platform provided by Google for online visual computing and analysis of global-scale geoscience data^30^. The platform mainly stores satellite images and other earth observation data, providing sufficient computing power to call and process the stored data. Compared with the Environment for Visualizing Images (ENVI), Earth Resources Data Analysis System (Erdas), and other traditional image processing software, the Google Earth Engine cloud platform can process images quickly and in batches without downloading image data. The platform provides online JavaScript API and offline Python API, and web services based on Google Cloud that can be quickly built by calling the API.

**Figure 2:**
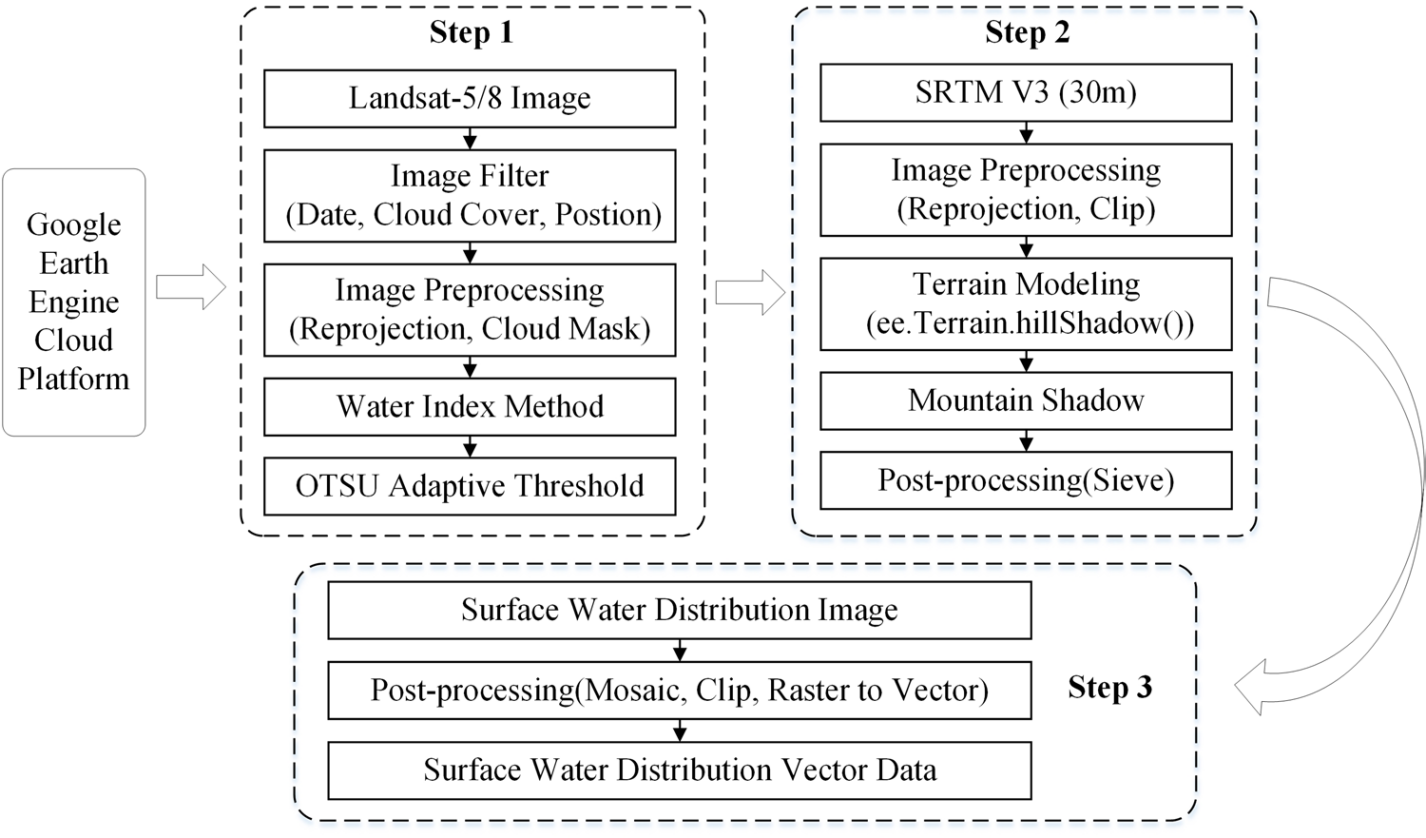
Rapid extraction model of surface water based on the Google Earth Engine.

To break the traditional remote sensing image water extraction mode and improve the efficiency and accuracy of obtaining surface water resource information distribution, a rapid extraction model of surface water based on the Google Earth Engine was constructed in this study. Figure 2 illustrates the process of model implementation. The first step was to select the appropriate Landsat-5/8 images based on the date, cloud cover, and location; perform reprojection and cloud mask processing; and obtain the surface water distribution image by combining the spectral water index method and OTSU algorithm. The second step included reprojecting and clipping the Shuttle Radar Topography Mission (SRTM) data^31^, identifying the mountain shadow by terrain modelling, and eliminating small patches through filtering. In the third step, the mountain shadow data obtained in the second step were used to mask the results of the surface water in the first step, eliminating the influence of mountain shadows on water extraction, and the final surface water distribution data were obtained by post-processing operations such as mosaicking, clipping, and raster to vector conversion.

## Results and discussion

### Comparison of spectral water index methods

**Figure 3:**
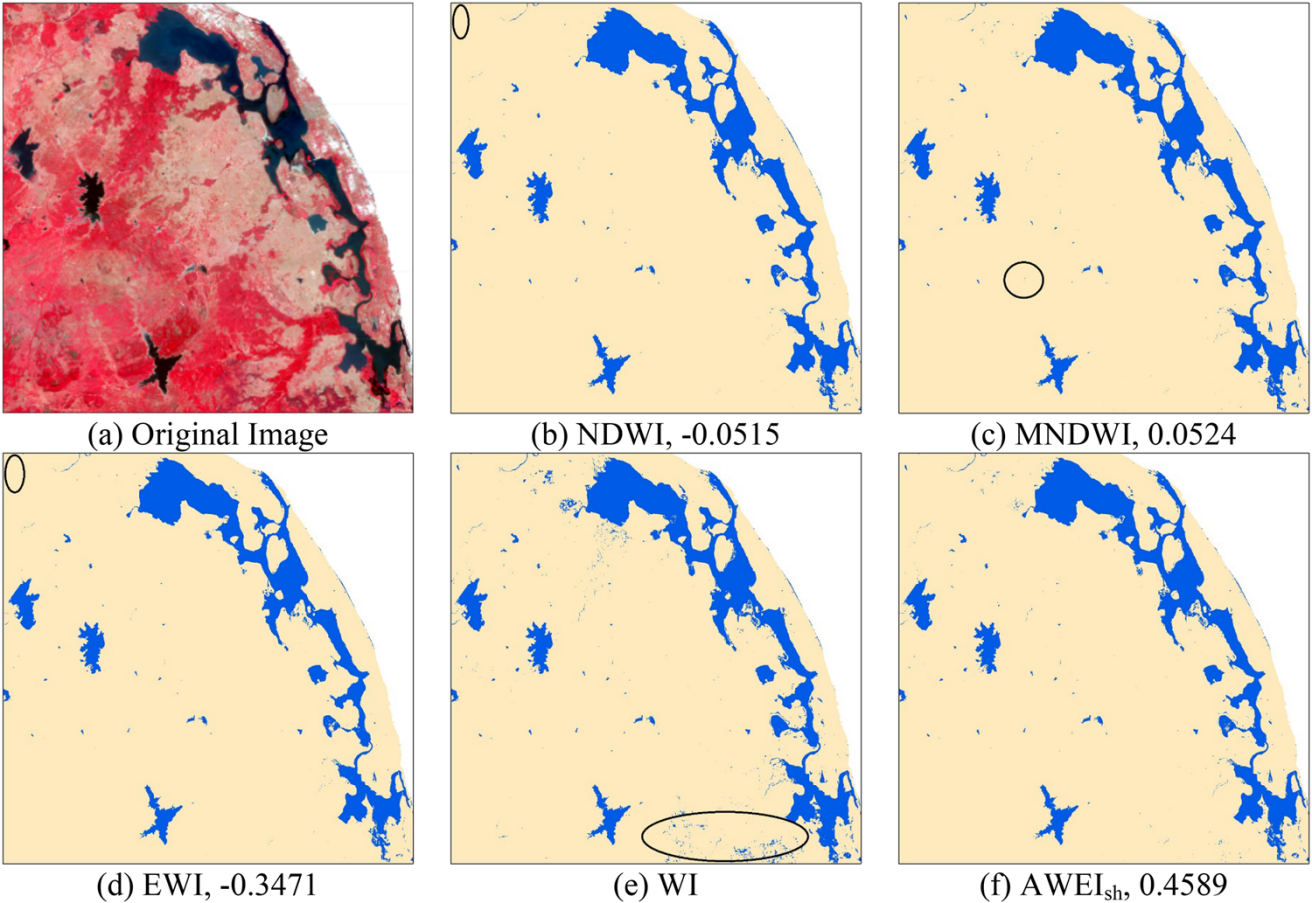
Results of water extraction from different spectral water index methods. (**a**) The original image. The threshold values and extracted water bodies from (**b**) NDWI, (**c**) MNDWI, (**d**) EWI, and (**f**) AWEI_sh_ methods determined by the OTSU algorithm. (*e*) The extraction result of WI.

Figure 3 shows the results of different spectral water index methods. Through overlay analysis with the original image and detailed visual analysis, it was found that AWEI_sh_ had the best extraction performance and could accurately identify the boundary of the water body. NDWI, MNDWI, and EWI had different degrees of leakage extraction; NDWI and EWI had an evident leakage extraction in the northwest corner of the image, and the water leakage extraction of MNDWI was mainly concentrated in the middle of the image. There was a lot of water body misidentified in WI, especially in the southeast corner of the image.

Based on the visual interpretation of the water boundary, 100 test samples were selected and the confusion matrix^32^ was calculated to obtain the extraction accuracy of the water body from three aspects: commission error, omission error, and overall accuracy (Table 1). As seen from the table, the overall accuracy of AWEI_sh_ was the highest, attaining a value of 99.14%, with extremely low commission and omission errors. WI had the lowest overall accuracy and the highest commission error, and could not distinguish water bodies and low reflectivity features effectively. The overall accuracies of NDWI, MNDWI, and EWI were similar. Comparing the results of the visual interpretation and quantitative analysis, the rapid extraction model of surface water based on the Google Earth Engine utilizing AWEI_sh_ index was used for assessing the spatiotemporal changes of water bodies.

**Table 1:** Accuracy comparison of different spectral water index methods.

Model	Omission error (%)	Commission error (%)	Overall accuracy (%)
NDWI	5.42	2.13	93.29
MNDWI	3.84	1.52	94.41
EWI	3.67	1.39	95.17
WI	1.54	13.25	86.13
AWEI_sh_	0.83	0.17	99.14

### Time series analysis of typical reservoir area

**Figure 4:**
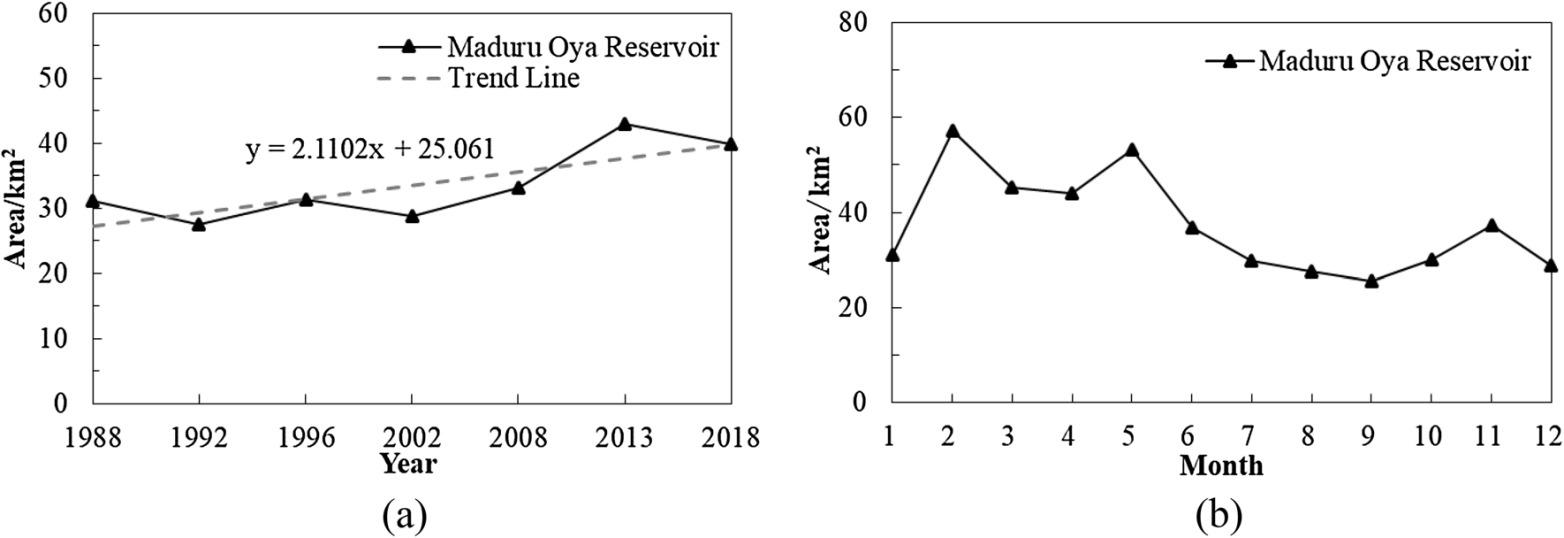
Observed area change in the Maduru Oya Reservoir. (**a**) Inter-annual variation of the Maduru Oya Reservoir area; (**b**) Intra-annual variation of the Maduru Oya Reservoir area in 2017.

To understand the inter-annual variation trend and intra-annual variation of the reservoir area in the dry zone of Sri Lanka, time series analysis was conducted with the Maduru Oya Reservoir as the case study area. The Maduru Oya Reservoir is the second largest reservoir in Sri Lanka, located in the east-central region, which is the main water source for irrigation and drinking, and has a high incidence of chronic kidney disease of unknown aetiology (CKDu). Figure 4 shows the inter-annual and intra-annual variations of Maduru Oya Reservoir area.

Figure 4 shows that the inter-annual fluctuation of Maduru Oya Reservoir area is slight, while the intra-annual fluctuation is significant. From 1988 to 2018, the reservoir area showed an overall increasing trend with slight float; the smallest area was recorded in 1992 (27.43 km^2^) and the largest area in 2013 (42.97 km^2^) (Fig. 4a). The rainy season in the dry zone of Sri Lanka occurs from October to February, and the dry season occurs from March to September. In 2017, the maximum area of the Maduru Oya Reservoir was noted in February, and the minimum area was noted in September. The area in February was 2.24 times bigger than that of September, with a difference of 31.58 km^2^. The maximum area of reservoirs or lakes generally occurs at the end of the wet season (February), and the minimum area occurs at the end of the dry season (September)^2^, which is consistent with the occurrence of maximum and minimum area in the Maduru Oya Reservoir in 2017(Fig. 4b). The area of the reservoir increased significantly in May during the dry season. According to meteorological data^33^, there were persistent strong winds and torrential rains in Sri Lanka in May 2017, resulting in an abnormal increase in the reservoir area. Generally, the period in which the area increased was from October to February (rainy season), while March to September (dry season) was the period in which the area decreased regardless of the influence of abnormal weather factors. The intra-annual fluctuation of the reservoir was severe, and there was a risk of drought and flooding at the same time. This observation implied that the seasonal regulation of water resources must be focussed in the future.

### Analysis of spatiotemporal change of inland lakes and reservoirs

**Figure 5:**
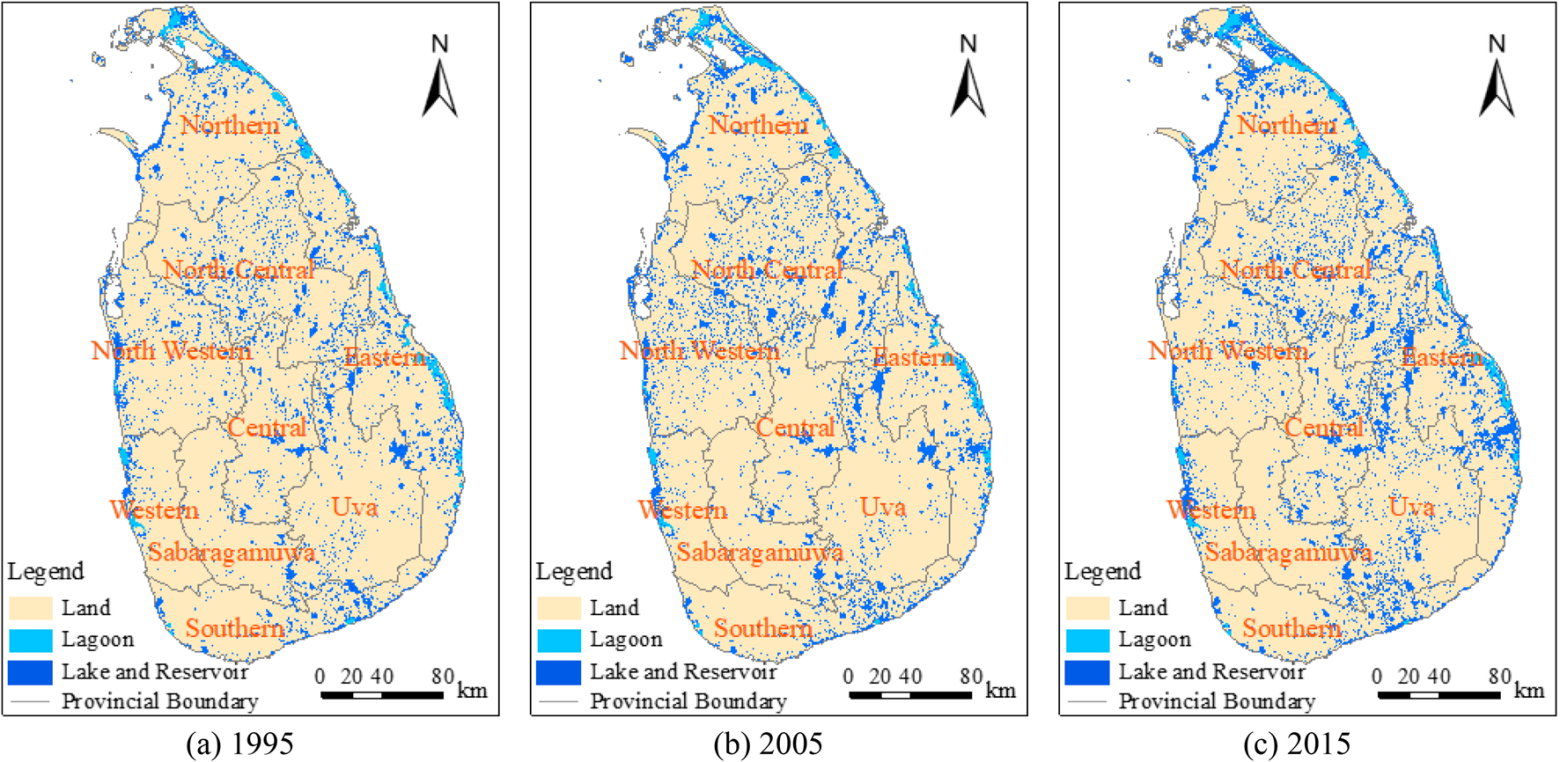
Water extraction results for Sri Lanka in 1995, 2005, and 2015. The administrative boundary data of Sri Lanka comes from the Humanitarian Data Exchange (HDX) open platform (https://data.humdata.org). The maps were generated by geospatial analysis of ArcGIS software (version ArcGIS 10.3; http://www.esri.com/software/arcgis/arcgis-for-desktop).

To systematically analyze the spatiotemporal variation characteristics of inland water in Sri Lanka in recent years, and considering the cloud cover of Landsat-5/8 images, 1995, 2005 and 2015 were selected as the study year with an interval of 10 years. The distribution information of surface water in three stages was obtained by running the rapid extraction model of surface water in the Google Earth Engine. According to statistics, the surface water areas of Sri Lanka in 1995, 2005, and 2015 were 1654.18 km^2^, 1964.86 km^2^, and 2136.81 km^2^, respectively. In the past 20 years, the water area of Sri Lanka has increased significantly. To further analyse the spatiotemporal changes of inland lakes and reservoirs, a 5-m buffer data of rivers in 2015 were produced in ArcGIS10.3 software; further, the area corresponding to the river channels were removed from the three images and only the lagoon areas were preserved. Lagoons are ubiquitous in the coastal areas of Sri Lanka, with flood discharge, aquaculture, coastal protection, and other functions^34^. The results consisting of the extracted lakes, reservoirs, and lagoons are shown in Fig. 5.

**Figure 6:**
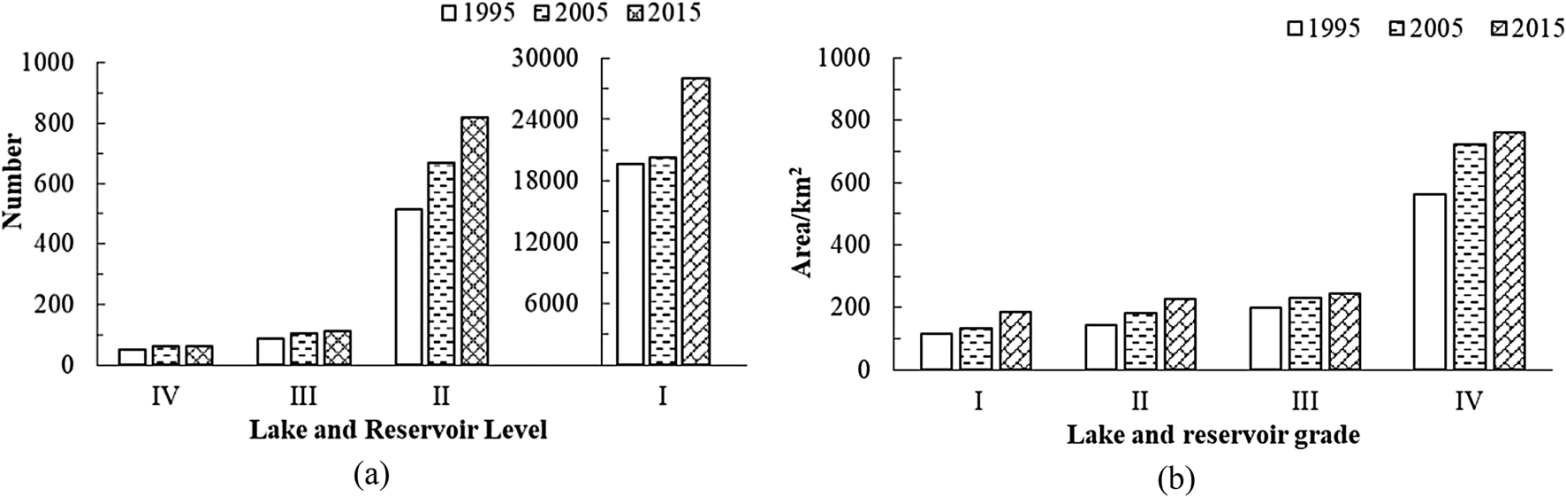
Number and area of lakes and reservoirs in Sri Lanka. (**a**) The number of lakes and reservoirs in 1995, 2005, and 2015; (**b**) Changes in lake and reservoir area in 1995, 2005, and 2015.

The overall water area of lakes and reservoirs in Sri Lanka showed an increasing trend from 1995 to 2015, and the lagoon area increased over these 20 years (Fig. 5). Because the lagoon does not belong to inland freshwater sensu stricto, the corresponding statistical analysis was not included in the following step. According to statistics, the total area covered of lakes and reservoirs in Sri Lanka were 1020.41 km^2^, 1270.53 km^2^, and 1417.68 km^2^ in 1995, 2005, and 2015 respectively. In the past 20 years, the area of lakes and reservoirs in Sri Lanka has increased by a considerable margin, attaining a value of 397.27 km^2^. To further analyse the spatiotemporal variation of inland lakes and reservoirs, they were divided into four grades according to their area: I (< 0.1 km^2^), II (≥ 0.1–1 km^2^), III (≥ 1–5 km^2^), and IV (≥ 5 km^2^). The number and area of different types of lakes and reservoirs for each year are shown in Fig. 6.

In Fig. 6a represents the number of lakes and reservoirs in the four grades, which showed an increasing trend from 1995 to 2015; the lower the grade of lakes and reservoirs, the greater the increase in area was observed. The number of I-grade lakes and reservoirs increased most significantly, while that of the IV-grade only increased by 11. Among the newly added IV-grade lakes and reservoirs, seven were transformed from other lakes and reservoirs, and four were newly built large reservoirs, such as the Rambukkam Oya, the Weheragala, the Daduru Oya, and the Mau Ara reservoirs. From 1995 to 2015, the area of the four grades of lakes and reservoirs showed an increasing trend, and the area of IV-grade lakes and reservoirs increased significantly with a total increase of 197.36 km^2^ (Fig. 6b). The higher the grade of lakes and reservoirs, the larger the total area. In 2015, the total area of IV-grade lakes and reservoirs was 760.53 km^2^, accounting for 54% of the total area among the four grades.

**Figure 7:**
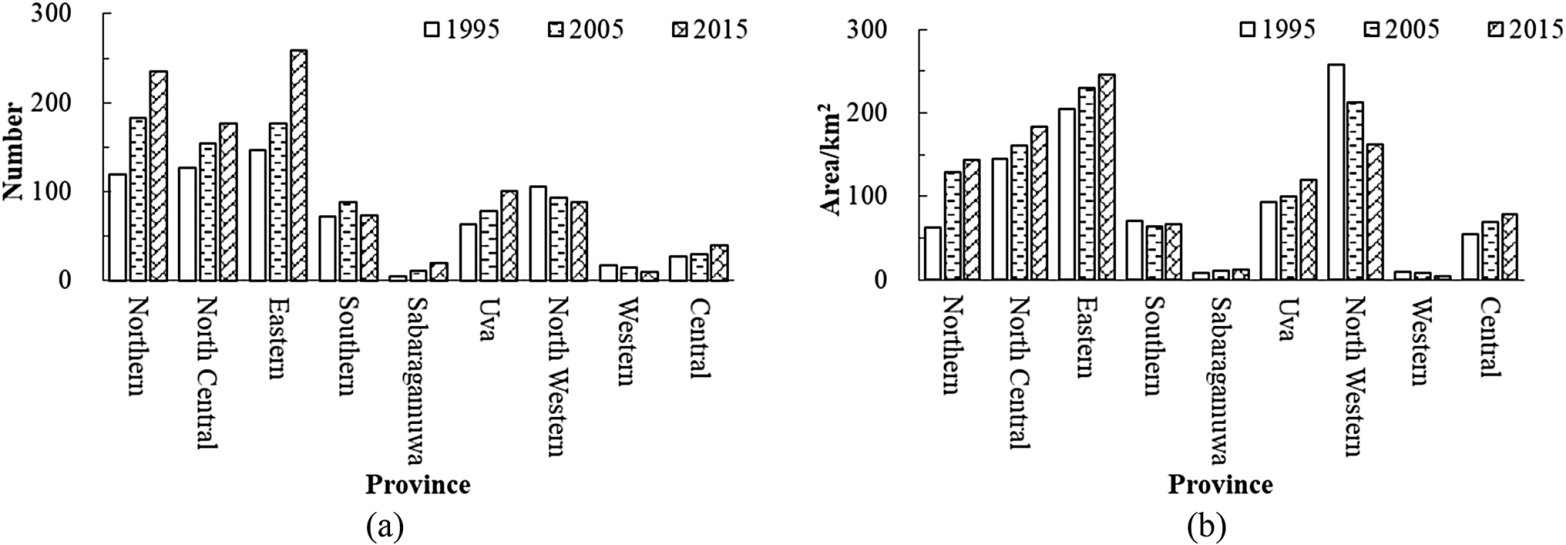
Number and area of lakes and reservoirs in each province in Sri Lanka. (**a**) The number of lakes and reservoirs in 1995, 2005 and 2015 in each province. (**b**) Changes in lakes and reservoirs area in 1995, 2005 and 2015 in each province.

Figure 7 shows the statistical results of the number and area of lakes and reservoirs in various provinces of Sri Lanka. From 1995 to 2015, the increase in the number and area of lakes and reservoirs in Sri Lanka were mainly concentrated in the dry zone, such as the Northern, North Central, Eastern, the Sabaragamuwa, the Uva, and Central provinces. The number and area of lakes and reservoirs in the Southern Province remained unchanged, whereas the number and area of lakes and reservoirs in the North Western and Western provinces decreased slightly. Nisansala et al. reported that the eastern, south eastern, northern, and north-central regions of the country experienced increasing rainfall trends from 1987 to 2017, while western regions and part of the northwestern and central regions of the country displayed a decreasing rainfall trend during the same period^35^. In recent years, Sri Lanka has built a large number of new water conservancy facilities to support agricultural irrigation, aquaculture, and local economic development, which can regulate the water distribution in the wet and dry seasons^36^. Therefore, in the provinces with the decrease of the number and area of lakes and reservoirs, the primary reason for the decrease was because of lesser amount of local rainfall. In the provinces with the increase of the number and area of lakes and reservoirs, the increase was mainly due to the increase in local rainfall and the construction of water conservancy facilities. In general, the number and area of lakes and reservoirs in the four grades differed, and the amount of available water resources in surface lakes and reservoirs in Sri Lanka showed an increasing trend.

## Conclusions

The optimal spectral water index method suitable for Sri Lanka was determined through experiments in this study. The spatiotemporal variation characteristics of the Maduru Oya Reservoir and inland lakes and reservoirs were analysed combining the index-based method with the rapid extraction model of surface water based on the Google Earth Engine. The primary conclusions of the study are as follows:

1. Compared with the four spectral water indices of NDWI, MNDWI, EWI, and WI, the overall accuracy of AWEI_sh_ was the highest (99.14%). AWEI_sh_ could accurately identify the surface water boundary, with extremely low commission and omission errors; therefore, it is suitable for the extraction of water bodies in Sri Lanka.
2. From 1988 to 2018, the Maduru Oya Reservoir area showed an overall increasing trend with small fluctuations. Compared with the inter-annual variation, the annual area changes of the reservoir in 2017 fluctuated significantly, and the maximum and minimum areas appeared in February and September, respectively. Regardless of the influence of abnormal weather factors, the area increased from October to February of the following year, and the area decreased from March to September. Hence, the management of water resources in the dry zone should focus on seasonal regulation and control.
3. From 1995 to 2015, the number and area of lakes and reservoirs of four grades in Sri Lanka increased in different degrees, i.e., the lower the grade of lakes and reservoirs, the greater the increase in the area of lakes and reservoirs. The increase in the number and area of lakes and reservoirs was mainly concentrated in arid provinces. Overall, the water resources of lakes and reservoirs in Sri Lanka showed an increasing trend.

The results of this study can act as a reference data and provide scientific support for water resource management and planning in Sri Lanka, providing an effective solution for rapid spatiotemporal variation analysis of surface water. However, there are still some shortcomings. Specifically, the extraction of the water body was based on Landsat images with 30 m resolution, and a water body with an area of less than 900 m^2^ may not be effectively extracted. Therefore, to achieve high-resolution water information and to perform spatiotemporal change analysis in Sri Lanka, Systeme Probatoire d'Observation dela Tarre (SPOT), Gaofen (GF) series, and other high-resolution images have to be used in future research.
